# Diagnosis of Ectopic Cervical Thymus in an Infant Using Noninvasive Imaging Methods

**DOI:** 10.7759/cureus.105826

**Published:** 2026-03-25

**Authors:** Marika Sugiyama, Tamaki Ikuse, Hiroyuki Sato, Hiroki Suganuma, Hiromichi Shoji

**Affiliations:** 1 Department of Pediatrics, Juntendo University Faculty of Medicine, Tokyo, JPN; 2 Department of Pediatrics and Child Health, Ikuse Clinic, Tokyo, JPN

**Keywords:** cervical mass, ectopic cervical thymus, ectopic thymus, magnetic resonance imaging, noninvasive diagnosis, pediatrics, starry-sky appearance, ultrasonography

## Abstract

Ectopic cervical thymus (ECT) is a rare congenital anomaly caused by the incomplete descent of thymic tissue during fetal development. Imaging modalities such as ultrasonography and magnetic resonance imaging (MRI) are useful tools for diagnosing this condition. However, ECT is often difficult to diagnose based solely on imaging findings because of its rarity. Surgical excision is frequently performed for differentiation from malignant neck tumors. We encountered a four-week-old male infant with left-sided neck swelling noted at three weeks of age. Ultrasonography revealed a well-defined hypoechoic lesion showing a fine mosaic-like internal pattern identical to that of the normal mediastinal thymus. MRI demonstrated a mass with signal intensity similar to the thymus, without airway or vascular compression. Based on these findings, ECT was diagnosed noninvasively, and the patient was managed conservatively, showing gradual regression over time. This case emphasizes that direct comparison of imaging characteristics between the cervical lesion and normal thymic tissue can make the diagnosis of ECT more definitive, thus allowing avoidance of invasive procedures and enabling safe conservative management.

## Introduction

Ectopic cervical thymus (ECT) is a congenital anomaly that occurs when part of the thymic tissue remains in the neck or fails to completely descend into the mediastinum during fetal development [1]. Although ECT is uncommon, it typically presents during infancy or early childhood as an asymptomatic cervical swelling. Most patients are asymptomatic, present with neck swelling only, and experience spontaneous regression during early childhood. In rare cases, large ECTs may cause airway obstruction or feeding difficulties in infants [2-5]. Despite its benign nature, ECT can be difficult to diagnose because its clinical and imaging features may mimic other pediatric cervical masses, including malignant tumors. Consequently, surgical excision is often performed to differentiate it from malignant cervical tumors [2,4,6]. In recent years, advances in imaging techniques have improved the ability to diagnose ECT noninvasively. Ultrasonography is particularly useful as a first-line modality because the thymus demonstrates characteristic internal echogenic patterns, often described as the "starry-sky" appearance, consisting of hypoechoic lymphoid tissue interspersed with echogenic septa and foci. Magnetic resonance imaging (MRI) can further support the diagnosis by demonstrating signal characteristics similar to those of the normal thymus. Here, we report a case of ECT that was noninvasively diagnosed by ultrasonography and MRI after neck swelling was noted during a one-month health checkup.

## Case presentation

A four-week-old male infant presented with swelling on the left side of the neck, which was first noticed by his parents at three weeks of age. There were no signs of respiratory distress or difficulty feeding. He was born at 41 weeks and one day of gestation (birth weight: 3654 g) via emergency cesarean section due to labor arrest. The Apgar scores were 1 and 7 at one and five minutes, respectively. No abnormalities were detected using fetal ultrasonography during pregnancy or the neonatal period.

**Figure 1:**
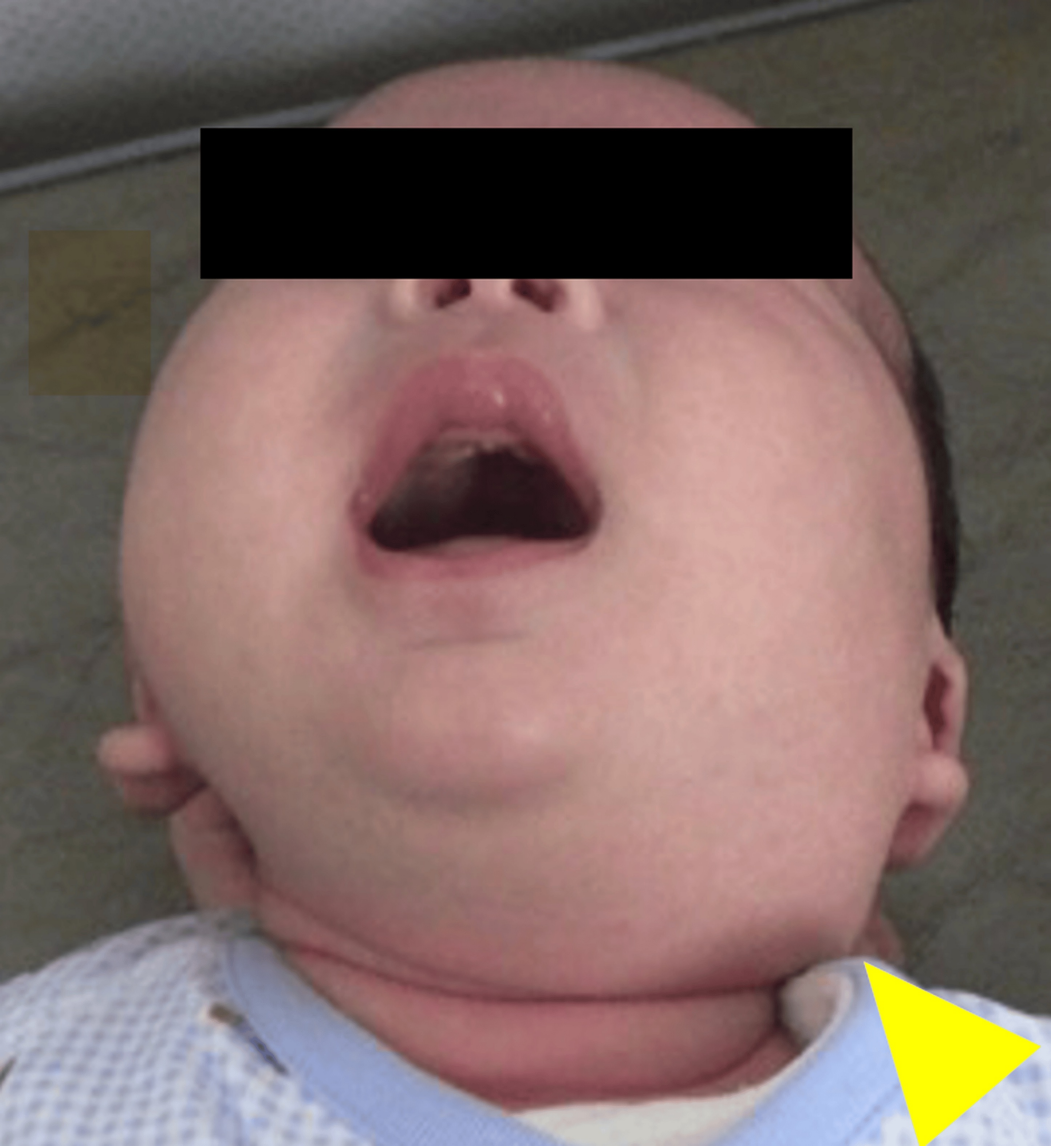
Appearance of the cervical lesion Neck swelling observed in the left cervical region (yellow arrowhead).

**Figure 2:**
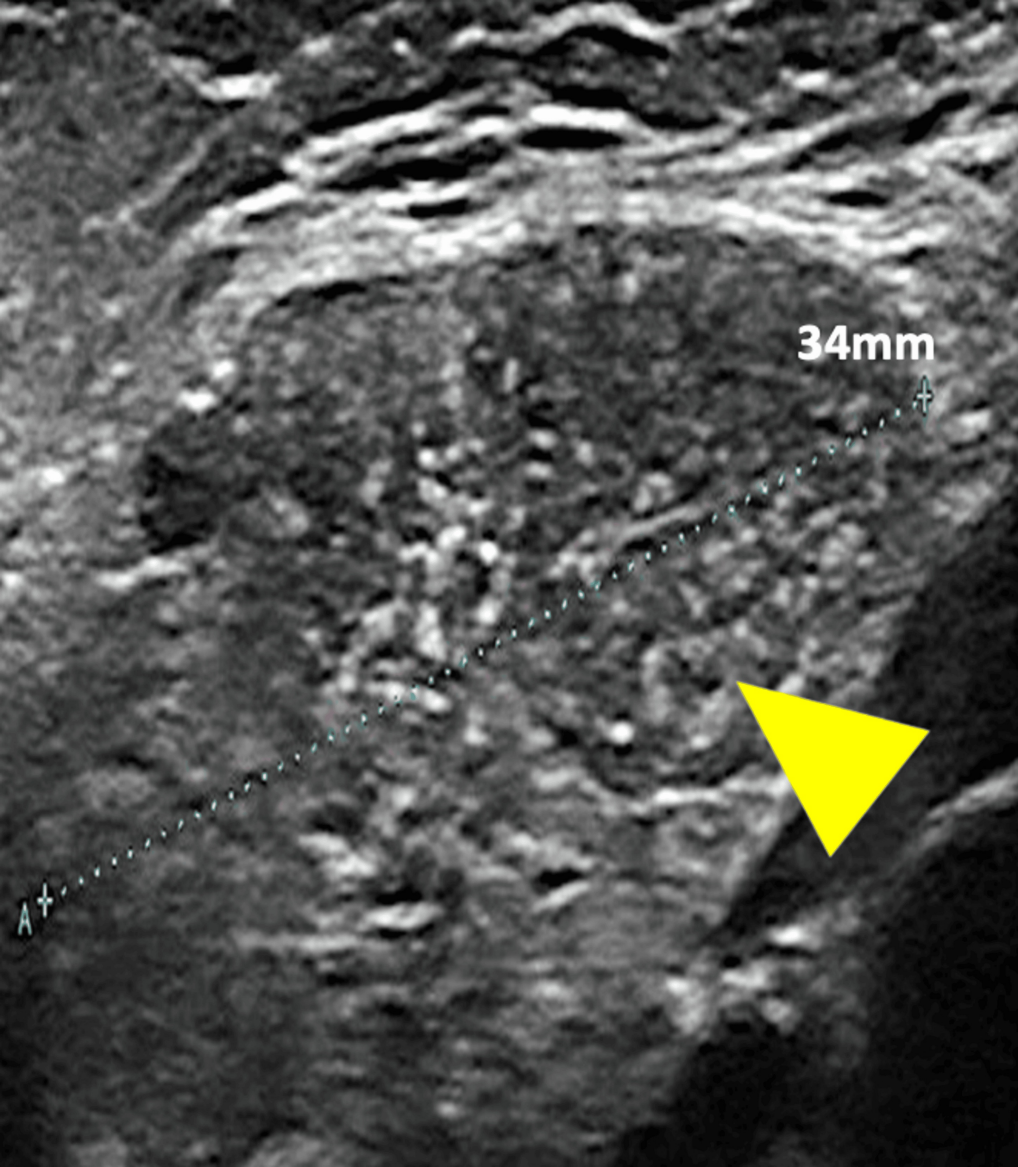
Ultrasound image of the cervical mass Ultrasound image (high-frequency linear probe, 11 MHz) of the cervical mass showing a well-defined hypoechoic mass measuring 34 mm in its longest diameter with a fine mosaic-like internal pattern of multiple branching echogenic linear structures and foci ("starry-sky" appearance) (yellow arrowhead).

**Figure 3:**
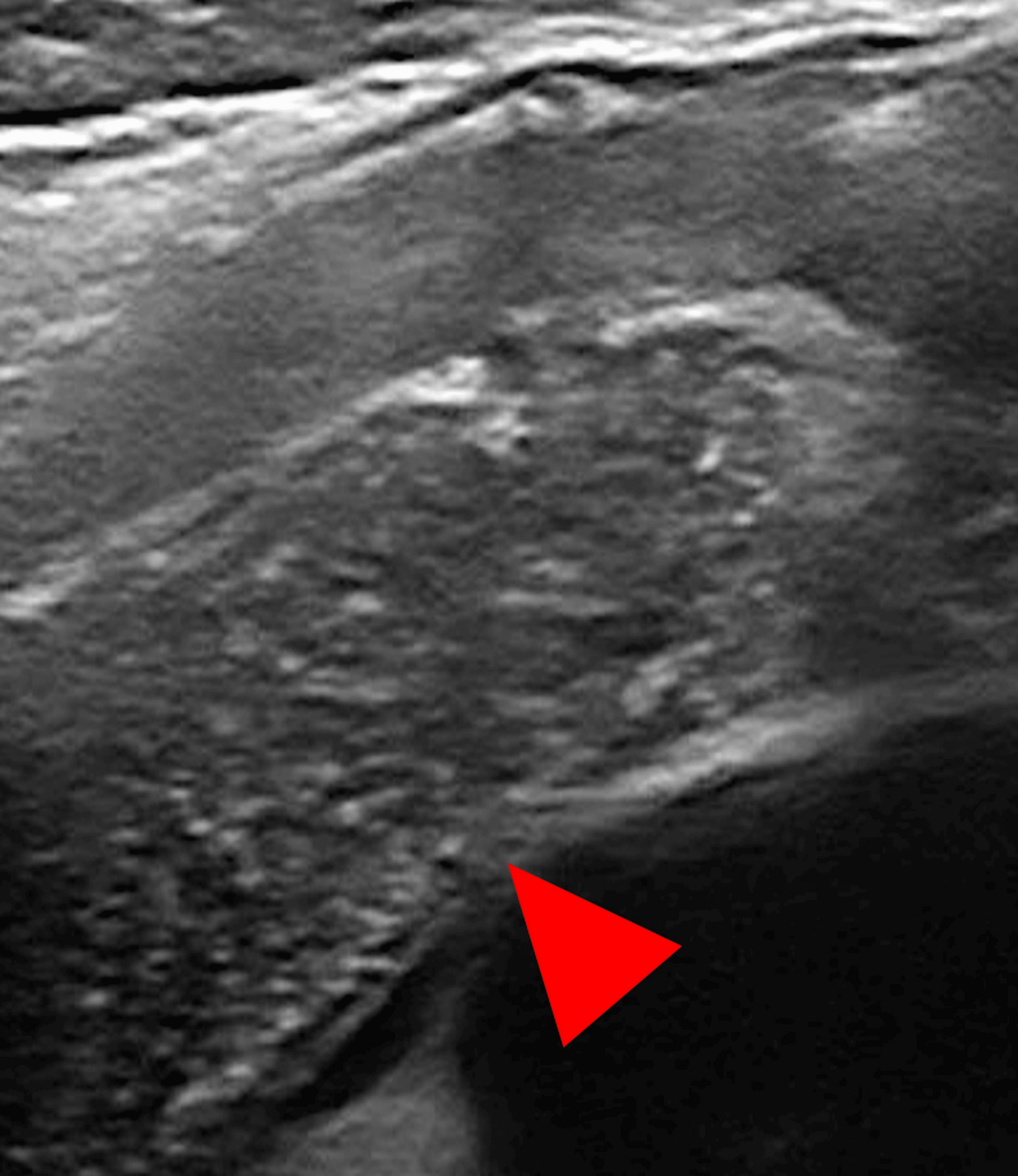
Ultrasound image of the thymus Chest ultrasound image of the normal thymus with an identical internal pattern (red arrowhead).

**Figure 4:**
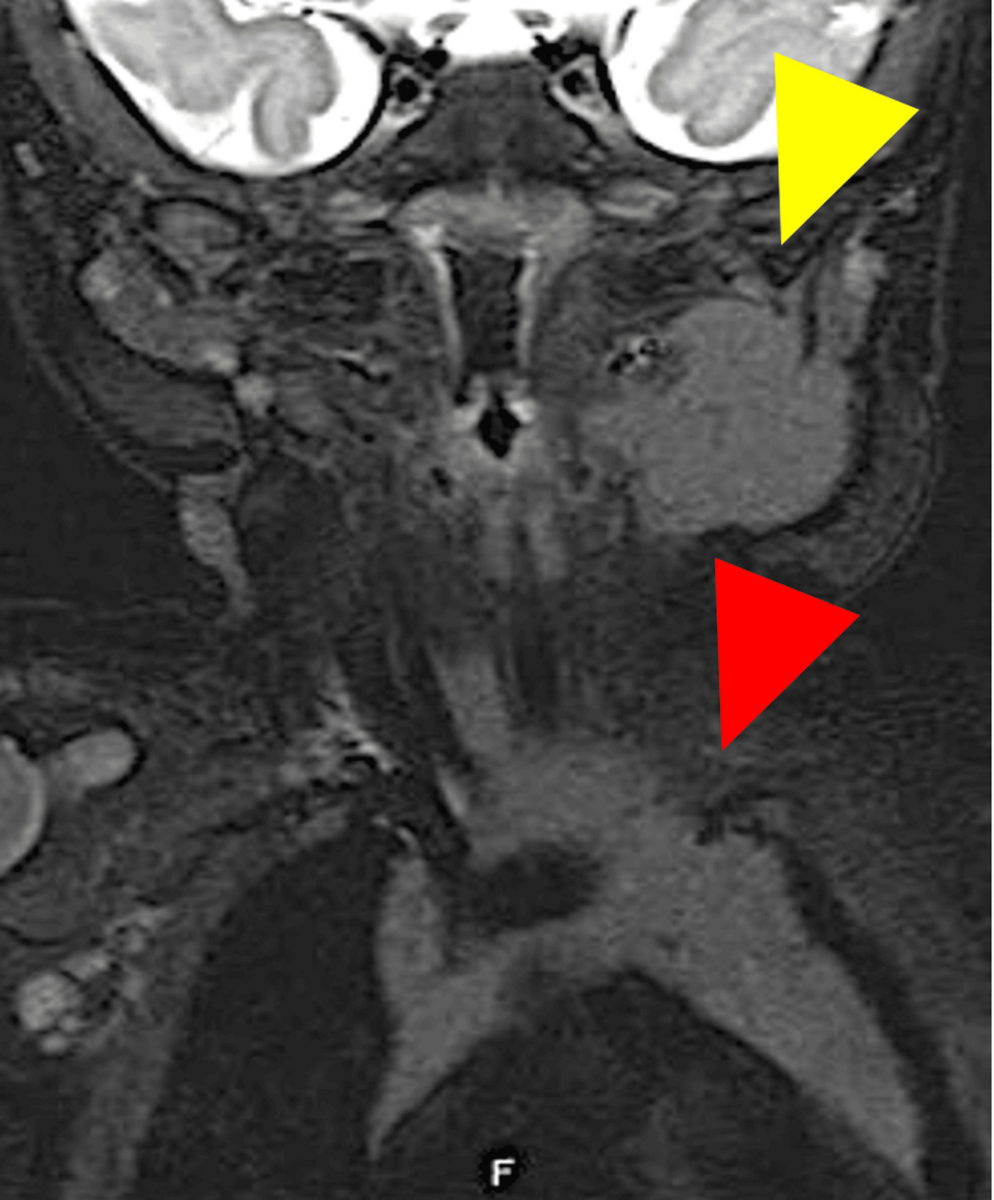
T2-weighted MRI of the cervical mass and the normal thymus T2-weighted MRI showing the cervical mass (yellow arrowhead) and the normal thymus (red arrowhead) with identical signal intensity. MRI: magnetic resonance imaging

Physical examination revealed soft, thickening of the subcutaneous tissue on the left lateral side of the neck with a fatty tissue-like consistency without a discrete palpable mass (Figure 1). No additional abnormalities were observed. The laboratory findings were within normal limits. Ultrasound examination revealed a well-defined, 34-mm hypoechoic mass containing branching echogenic septa and punctate echogenic foci, producing a characteristic "starry-sky" appearance (Figure 2). Color Doppler imaging revealed no abnormal internal vascularity and no compression of adjacent vessels. The lesion extended deep to the sternocleidomastoid muscle. Anechoic areas suggesting cystic structures, such as those seen in lymphatic malformations or branchial cleft cysts, were not identified. No features suggestive of malignancy were observed, including a heterogeneous internal echo pattern, ill-defined margins, vascular proliferation, infiltration into surrounding tissues, or structural abnormalities in the surrounding lymph nodes. To compare this imaging characteristic with normal thymic tissue, a chest ultrasound was performed, revealing a normal thymus in the anterior mediastinum exhibiting an identical internal echogenic pattern (Figure 3). MRI revealed that the mass was isointense on T1-weighted images and hyperintense on T2-weighted images, extending from the retropharyngeal space to the parapharyngeal space without airway or vascular compression. The cervical mass and normal thymus exhibited identical imaging characteristics across all imaging modalities (Figure 4). These findings facilitated the diagnosis of ECT.

The differential diagnoses associated with cervical masses in infants include branchial cleft cysts, lymphatic malformations, hemangiomas, hamartomas, thyroglossal duct cysts, teratomas, lymphomas, neuroblastomas, and dermoid cysts. However, none of these conditions matched the clinical or imaging features observed in this case. Additionally, low levels of urinary vanillylmandelic acid and homovanillic acid led to the elimination of neuroblastoma as a possible diagnosis.

Follow-up ultrasonography was performed every 2-3 months to monitor changes in the mass size and confirm the absence of airway or vascular compression. No change in size was observed until eight months of age, after which the mass began to gradually decrease. At two years of age, the mass measured 28 mm at its longest diameter, with no compression or associated symptoms.

## Discussion

The thymus plays a vital role in immune system development, arising from the ventral portion of the third pharyngeal pouch by the fifth week of gestation and descending into the anterior mediastinum by the seventh week [7]. ECT is the result of incomplete descent during this process. Although rare, with an estimated prevalence of 0.99-1.8% [8], an autopsy study has reported that the incidence of ECT is as high as 31% in children [9]. 

Traditionally, histopathological evaluation using fine-needle aspiration cytology or biopsy has been required for a definitive diagnosis [2]. However, imaging-based diagnosis has become prominent owing to its noninvasive nature. Ultrasonography is the first-line diagnostic tool and depicts the thymus as a starry-sky appearance [10]. MRI further supports the diagnosis by showing isointensity on T1-weighted images and hyperintensity on T2-weighted images compared to the intensity of the surrounding muscle. Despite these advances, previous systematic reviews have reported that imaging alone only yields a correct preoperative diagnosis in 16.9% of cases, highlighting the diagnostic challenges associated with this rare entity [2].

At our institution, there have been no prior instances of imaging-based diagnosis of infant ECT. Therefore, careful differentiation from other types of cervical masses was essential. The key to diagnosis is the direct comparison of imaging characteristics between the cervical mass and the normal thymus. This approach excludes other differential diagnoses and enhances diagnostic confidence, thereby avoiding unnecessary surgery and associated complications. However, a limitation of this case was the absence of histopathological confirmation [5]. A definitive diagnosis of ECT is only possible through histopathological examination, meaning that imaging diagnosis of ECT carries a slight risk of missing malignant disease. Therefore, to manage ECT conservatively, regular ultrasound examinations are necessary to confirm the following: no compression of the trachea or blood vessels, no emergence of signs suggestive of other malignant tumors, such as tumor enlargement or heterogeneous internal structure, and no observed deviation from the echographic findings of a normal thymus. If any of these findings are observed, further evaluation should be considered. Ultrasound follow-up should be tailored according to patient age and changes in lesion size. Shorter intervals (e.g., every 2-3 months) may be considered initially, with extension to yearly examinations once a reduction in lesion size is confirmed [11]. Long-term follow-up is necessary to ensure the complete regression of the mass and exclusion of other potential diagnoses. Nonetheless, no other known cervical masses in infants demonstrate identical imaging features or a spontaneous regression pattern similar to that of an ECT, further supporting the diagnosis.

## Conclusions

This case highlights the importance of considering ECT as a differential diagnosis of cervical masses in infants. Noninvasive imaging studies, particularly ultrasonography and MRI, can provide highly suggestive evidence for ECT when compared with normal thymic imaging features and may allow avoidance of invasive diagnostic procedures. Conservative management with periodic imaging may be appropriate for this benign condition.
